# Development of a functional electrical stimulation cycling toolkit for spinal cord injury rehabilitation in acute care hospitals: A participatory action approach

**DOI:** 10.1371/journal.pone.0316296

**Published:** 2025-02-10

**Authors:** Hope Jervis-Rademeyer, Srijana Gautam, Stephanie Cornell, Janelle Khan, Danielle Wilanowski, Kristin E. Musselman, Vanessa K. Noonan, Dalton L. Wolfe, Riccardo Baldini, Steven Kennedy, Chester Ho

**Affiliations:** 1 Department of Medicine, University of Alberta, Edmonton, Alberta, Canada; 2 Parkwood Institute, St. Joseph’s Health Care London, London, Ontario, Canada; 3 Lawson Health Research Institute, London, Ontario, Canada; 4 Royal Alexandra Hospital, Alberta Health Services, Edmonton, Alberta, Canada; 5 University of Alberta Hospital, Alberta Health Services, Edmonton, Alberta, Canada; 6 Department of Physical Therapy and Rehabilitation Sciences Institute, Temerty Faculty of Medicine, University of Toronto, Toronto, Ontario, Canada; 7 KITE Research Institute, Toronto Rehabilitation Institute-University Health Network, Toronto, Ontario, Canada; 8 Praxis Spinal Cord Institute, Vancouver, British Columbia, Canada; 9 University of Western Ontario, London, Ontario, Canada; 10 University of Alberta, Edmonton, Alberta, Canada; 11 Glenrose Hospital, Edmonton, Alberta, Canada; University of Illinois Urbana-Champaign, UNITED STATES OF AMERICA

## Abstract

The purpose of our study was to develop a toolkit to facilitate the implementation of functional electrical stimulation (FES) cycling for persons with a newly acquired spinal cord injury (SCI) in the acute care inpatient hospital setting. The researchers and community members used participatory action as a research approach to co-create the toolkit. We held two focus groups to develop drafts, with a third meeting to provide feedback, and a fourth meeting to evaluate the toolkit and determine dissemination strategies. Toolkit development followed the Planning, Action, Reflection, Evaluation cycle. We used an iterative design informed by focus group and toolkit consultant (SC) feedback. In focus group discussions, we included FES cycling champions (JK, DW) who led acute care implementation. Focus group members, recruited through purposive sampling, had to 1) have an understanding about FES cycling in acute care for SCI and 2) represent one of these groups: individual living with SCI, social support, hospital manager, clinician, therapist, researcher, and/or acute care FES cycling champion. Twelve individuals took part in four focus groups to develop a toolkit designed to facilitate implementation of FES cycling in SCI acute care in Edmonton, Alberta. Group members included an individual with lived experience, three acute-care occupational or physical therapists, three acute-care hospital managers, and five researchers. Two physical therapists also identified as clinical FES cycling champions. Following an inductive content analysis, we identified four main themes: 1) Health care provider toolkit content and categories, 2) Health care provider toolkit end product, 3) Collaborations between groups and institutions and 4) Infrastructure. Interested parties who utilize FES cycling in acute care for SCI rehabilitation agree that toolkits should target the appropriate group, be acute care setting-specific, and provide information for a smooth transition in care.

## 1. Introduction

Activity-based therapy (ABT) after spinal cord injury (SCI) involves repetitive, task-specific movement practice that stimulates the spinal cord below the level of the lesion [1]. Technology can be used to facilitate the high dosage that is required for ABT [2]; for example, increased movement repetitions (e.g., number of strides and speed when walking) or peak change in heart rate [3]. Functional electrical stimulation (FES) cycling for the lower extremities is a type of ABT. During FES cycling, an electrical current is applied to lower body muscles to facilitate patterned leg cycling movement in those with muscles with impaired function caused by conditions such as SCI. Earlier clinical studies testing FES cycling for SCI rehabilitation in the subacute phase after SCI (i.e., post-emergency care) have demonstrated promising results (e.g., prevented loss of muscle mass [4], improved muscle power [4], decreased rate of bone mineral density loss(5)). Across both studies, participants who were in the intervention group (*n = 17*) ranged from a C5 to T8 level of injury and most (*n = 16*) had a neurologically complete SCI motor lesion (American Spinal Injury Association impairment scale (AIS) grade A). The remaining participant was rated AIS B [4,5]. As part of current clinical practice, health care providers (HCPs) do not apply ABT, like FES cycling, soon after SCI [6]. This indicates a mismatch between what peer-reviewed literature would suggest and clinical practice. Recognizing the need to improve ABT access and quality for individuals living with SCI, the Canadian ABT Community of Practice (ABT CoP) was formed [7]. The ABT CoP is a collaborative network of groups interested in ABT. However, despite these initiatives and research findings, FES cycling is not widely used as a rehabilitation therapy during the acute stage of SCI in Canada.

There has been a significant increase in peer-reviewed published research according to PubMed searches for *SCI and FES*, including the subtopic *FES cycling*, over time. However, it can take up to 17 years for research to be adapted into clinical practice [8–10]. Barriers to using technology (e.g., FES cycling) in clinical practice include low confidence in technology for both patients and therapists [11–16]. During acute SCI, patients are undergoing numerous physical challenges (e.g., respiratory, temperature regulation) [17]. They may be learning different ways to move, function, or how to use a device, like a wheelchair. Patients also report an emotional adjustment, which may inhibit their ability to retain information, or to engage in lengthy discussions [18]. Together, these factors may affect a patient’s ability to incorporate a new technology into their rehabilitation even with the support of a clinician. Clinicians report limitations in device knowledge including safety requirements and a lack of practical skills [19]. A steep learning curve and extensive instructions paired with time constraints affect technology use. Clinicians must face these time constraints, staff shortages, and meet discharge goals, which compete with therapy goals (e.g., providing ABT) [20]. These factors may affect the introduction of rehabilitation technology. Also, the rehabilitation technology must be integrated with service delivery and align with patients’ rehabilitation goals [19].

Toolkits have been designed to improve the uptake of rehabilitation knowledge (e.g., assessments, interventions). Moreover, the implementation of these toolkits has increased the use of the interventions (e.g., exercise programs, technologies) they targeted [21]. For example, Tyson et al. developed a toolkit for stroke rehabilitation which significantly increased the number of uses of all standardized measurement tools from 36% to 81% in clinical team meetings [21]. A toolkit can also provide a means to communicate with clinicians [22]. It can be used to advocate for the intervention as an evidence-based toolkit provides an extra layer of credibility. To boost the implementation of rehabilitation strategies like toolkits into clinical practice, champions are a key component [23,24]. These clinical champions can enhance stakeholder engagement, provide operational knowledge, model discipline adherence and a team approach to implementing strategies, and help to problem solve individual barriers to use.

Patients, HCPs, and administrative staff have described some benefits of toolkits according to their perspectives. In a study by Wallace et al., patients noted that toolkits offer a centralized spot for accurate and relevant information, reducing the need for an online search. They would like personalized information within toolkit. Toolkits may be especially useful for vulnerable populations (e.g., older adults, homeless, financially unstable), or simply those people who live alone, or do not speak English [25]. Additionally, HCPs have provided positive feedback about implementing toolkits, such as facilitating communication with patients and families, informing planning and decision-making, and improving staff camaraderie [21]. Administrative staff have suggested that toolkits can reduce the staff burden, particularly if they are self-directed and easy-to-use [25].

Currently, clinical training, ongoing support, and resources are typically provided by companies that sell FES cycles. This training is specific to the type of equipment (e.g., FES cycle), yet it may not consider contextual nuances (e.g., stage of injury, type of setting, workflow, work processes). As documented in a recent study by Jervis-Rademeyer et al., acute care therapists who work with individuals with SCI identified themselves as ‘passionate groups’ receptive to integrating ABT (e.g., FES cycling) [6]. Therapists who took part in the study desired educational materials tailored to the acute care setting, which currently do not exist for FES cycling in Canada. According to a clinical practice guideline by Fehlings et al., it is suggested that rehabilitation should be  offered to patients with acute SCI when they are medically stable and can tolerate the necessary rehabilitation intensity [26]. This means that therapies can be started before patients are transferred from the acute care setting to inpatient rehabilitation. Furthermore, research has shown that FES cycling has been implemented 2–3 weeks post-SCI [27,28]. The median hospital (i.e., emergency and acute care) length of stay in Canada from 2011 to 2014 was 30 days prior to moving to inpatient rehabilitation where 78 days was the median length of stay for someone with SCI [29]. These statistics indicate that there is enough time to start an FES cycling program, even by providing related educational resources for patients, during acute care.

Edmonton, Alberta has two SCI trauma centres (Royal Alexandra Hospital and University of Alberta Hospital) that provide acute care for individuals with SCI. These hospitals also have FES cycles or pending deliveries of the equipment. FES cycles for SCI rehabilitation are in use at the rehabilitation and community settings in Edmonton. Together, these findings suggest an opportunity to create a toolkit to assist with FES cycling implementation in acute care in Edmonton, Alberta to connect the continuum of care with the potential for future expansion.

### 1.1. Purpose

Our study aim was to develop a toolkit to facilitate the implementation of FES cycling during the acute stage of SCI rehabilitation.

### 1.2. Study objectives

The objectives of the proposed project were to 1) identify and prioritize evidence-based information to facilitate the implementation of FES cycling for persons with a new SCI in acute care, and 2) develop a FES cycling toolkit specific to acute SCI rehabilitation given prioritization results.

## 2. Methods

### 2.1. Design

We used a qualitative descriptive design. To address Objectives 1 and 2, we used a participatory action research (PAR) methodology that enabled us to identify priorities for the toolkit [30]. To address Objective 2, toolkit development followed the Planning, Action, Reflection, Evaluation cycle, with evaluation performed after the final version of the toolkit was complete.

### 2.2. Participants and setting

Focus group participants were approached by the first author (HJ-R) via email using purposive sampling from 23 October 2022 to 17 March 2023. Although some focus group members contributed to the research process (i.e., research team), some individuals were only part of the focus groups. Therefore, we refer to focus group members as participants. There were some prior relationships between the research team and the participants as the SCI community is small with some individuals belonging to the same groups. Participants had experience or understanding of FES cycling in acute care for individuals living with SCI and represented one of the following groups: individual with SCI, hospital manager, therapist (e.g., occupational or physical therapist), researcher, and/or acute care FES cycling champion. Focus group members were deidentified on transcripts and published materials.

Survey participants attended the FES cycling toolkit presentation at the Royal Alexandra Hospital or the University of Alberta Hospital. Individuals who participated in the toolkit usability survey were anonymous and data was encrypted through SurveyMonkey. Written informed consent was obtained from all participants.

To calculate the sample size for our qualitative study we used the concept of ‘information power’ [31]. This concept is based on an evaluation of the study’s aim, sample specificity, quality of interview dialogue, analysis strategy, and use of established theory. According to our assessment of these factors, our study holds a large amount of information power, thus requiring a small sample size. Our study employed focus groups, which can consist of up to 12 individuals [32,33].

Ethics for this study was obtained from the University of Alberta Health Research Ethics Board and Alberta Health Services Operational Approval was granted for the University of Alberta Hospital and the Royal Alexandra Hospital. This paper adhered to reporting standards according to the COnsolidated criteria for REporting Qualitative Research (COREQ) checklist [34].

### 2.3. Data collection and analysis

Four focus groups were used to collect data to develop the toolkit. We held two focus groups to develop drafts, with a third meeting to provide feedback. One focus group meeting occurred after the final toolkit draft was created for evaluation and discussion of dissemination strategies. Focus group discussions were based on a semi-structured interview guide (S1 Appendix). The focus group facilitator (HJ-R) is an experienced qualitative interviewer with a physical therapy background. At the time of the study, she was a postdoctoral fellow. We used an iterative design informed by focus group feedback. A toolkit consultant (SC) and co-investigator (DLW) from Parkwood Institute provided toolkit guidance because of their expertise in toolkit development [35]. As part of our project, we included FES cycling champions already embedded within acute SCI care. In turn, these champions delivered coworker’s perspectives to the focus groups. Champions also educated HCPs within their departments about the toolkit and advocated for its use. Data were collected through audio recordings of focus group discussions via the Zoom platform. These data were transcribed using Rev.com which were reviewed by HJ-R and SG. Coding was managed using MS word due to the amount of data.

After presentations of the draft toolkit at the Royal Alexandra and University of Alberta Hospitals, attendees were provided with a link to fill out a short online usability survey based on the System Usability Scale (SUS) (S2 Appendix) [36]. The SUS is used to collect a usability evaluation of a system (e.g., website). According to an evaluation by Lewis, the SUS has high reliability and concurrent validity with other similar measures [37]. According to survey results we made technical modifications to the toolkit. Survey data were collected through Survey Monkey.

We used an inductive content analysis to describe the focus group data about FES cycling in acute care for SCI rehabilitation [38]. Two members of the research team (HJ-R, SG) independently immersed themselves in the data by re-reading and annotating transcripts. From there, they developed concise labels and grouped them into categories. Then they met with a third researcher (CH) to finalize labels and categories. A researcher with a physical therapy background (DE) and an occupational therapist with a background in rehabilitation technology (KB) reviewed categories and representative quotes to ensure all categories were captured in the analysis. Results from each of the previous focus groups were discussed in the following focus group with participants. For example, the results from focus group 1 were discussed within focus group 2 and so forth.

## 3. Results

Four focus group meetings were held with participants (*n* = 12, 5M:7F) from the following groups: persons with lived experience (*n* = 1), hospital managers (*n* = 3), researchers (*n* = 5), and physical and occupational therapists (*n* = 2, *n* = 1). Two physical therapists were also identified as clinical champions (JK, DW). Four researchers had clinical backgrounds. These focus groups lasted from 45 to 59 minutes and took place from March to December 2023. Seven anonymous respondents who self-identified as health care providers completed the toolkit usability survey (Table 1). One participant skipped all questions on the survey. The average toolkit usability score is 68. Our toolkit usability score was 67.9 indicating that the usability of the website needed improvement.

As part of the inductive content analysis, we identified four main categories. 1) HCP toolkit content and categories, 2) HCP toolkit end product, 3) Collaborations between groups and institutions, and 4) Infrastructure (see Table 2).

**Table 1:** Functional electrical stimulation cycling toolkit website system usability scale survey results.

Respondent	Usability score/100
P1	60
P2	70
P3	55
P4	82.5
P5	87.5
P6	70
P7	50
P8	No responses given
Average usability score/100	67.9

**Table 2:** Categories, subcategories, and illustrative quotations from focus group participants.

Category and subcategories	Quotation number	Illustrative quotations
1. Health care provider toolkit categories and content		
a. Overall considerations	Q1	*“I guess if I think about implementations for use there, we sort of think about would it, like, who is the end user? So we have clinicians that make the decisions on selecting FES cycling. And then we have our group of assistants who, um, they, they log the patient in, they sort of make, they run the actual session. And so the knowledge base is slightly different than what our therapists will need. Um, and then when we have students kind of floating in, they’re very hands on with that hardware technical setup.” -P5 (Res)*
	Q2	*They should be different...with some different categories with input from the groups to identify specifically how important categories are…tailoring to each group is important.” -P5 (Res)*
b. Introduction	Q3	*“In addition to all these categories it might be useful to have an introduction, um, and like just some context setting, um, that might touch on some of these but at a very high level.” -P5 (Res)*
	Q4	*“Rationale for staff for why we should incorporate this into acute care…” -P2 (Res)*
	Q5	*“I think some wording around the evidence that we know about FES cycling and expectations around recovery. And having that somewhat standardized or at least clear for the therapist.”- P9 (Res)*
c. Benefits and risks	Q6	*“I think benefits and risks.”- P4 (Admin)*
d. Bike login, setup, and modifications	Q7	*“it’d be really helpful to have even the setup of the bike, all the information regarding like what proper setup, you know, distance, how you can modify, um, depending on, like, the seat heights and whatnot, um, that we learned in our training session.”—P7 (OT)*
	Q8	*“the logistics around even the setup of the, like, how to log into the bike. What do you do on your computer beforehand? What is the prep you need to do to get a patient profile up? “-P4 (Admin)*
e. Dosage parameters, muscle selection and outcome measures	Q9	*“Just like a quick cheat sheet almost so that it makes it a little bit quicker for clinicians to just take that and then start with those parameters and they can modify from there if they need to.”-P6 (PT)*
	Q10	*“Parameter selection, muscle selection (and) how to facilitate decisions” -P8 (Res)*
	Q11	*“Some functional measures of sort of that mix of, of agreed upon outcome measures for, um, the cycling would be great, especially as we move towards standardized data collection and trials.”—P9 (Res)*
f. Patient screening	Q12	*“I had most of the same kind of things like the precautions, contraindications, indications.”—P6(PT)*
	Q13	*“I think a, a bit of all of that will have to come into play. Um, especially like even medical stability ‘cause if we’re looking at early on, um, in the acute phase, um, are they ready, uh, are they able to participate? And yeah, the emotional side and, and the physical side, just the whole, I guess.” -P3 (Admin)*
g. Before, during, and after the FES cycling session	Q14	*“How to prepare and things to do after, like (a) before and after checklist, maybe.” -P4 (Admin)*
	Q15	*“Maybe half an hour before the treatment session, like these are the things that need to be checked. And then again, post session, um, having nurses or the care team kind of checking vitals and doing skin checks and so that pre and post kind of checklist as well for the healthcare providers we thought would be a helpful tool.” -P6 (PT)*
	Q16	*“I guess when you’re in the appointment versus things to look out (for) immediately after, or the next day.” -P4 (Admin)*
h. Glossary	Q17	*“So I think that [definitions related to FES parameters], we can leave it in, but other medical definitions, I think [health care providers] should know.”—P10 (PT)*
2. Health care provider toolkit end product		
a. PDF	Q18	*“I did send a, a paper just to [Moderator], um, that is some of the evidence behind why PDFs work well, but I think ‘cause they’re easily shared, easily posted.” –P8 (Res)*
	Q19	*“I think my understanding with a PDF format for a toolkit is links to those multimodal aspects are all embedded. So it isn’t just a PDF, it really is more of an interactive. Takes you to a quiz where you can test your knowledge, takes you to a video to demonstrate the application of something. “– P8 (Res)*
b. Website	Q20	*“Like for healthcare professionals, I think like the website with like all the multimedia links, personally like in my like, this is just my opinion that I think that’s enough.”—P7 (OT)*
	Q21	*“I’m just thinking with apps like that, most of us don’t have work phones. So then you’re expecting people to download this onto their personal phone, which I don’t know if a lot of people will necessarily want to do.”—P7 (OT)*
	Q22	*“And in some prep research, I did for the toolkit projects, therapist told us, did like a visual info, pictures, videos, and case examples.”—P8 (Res)*
3. Collaboration between groups and institutions		
a. Local context	Q23	*“I’m just thinking one thing, I don’t know if this is something you guys are already doing is maybe considering, kind of like a soft launch, but like sending the toolkit to some of the therapists that do more FES cycling and have more experience with it.”—P7 (OT)*
	Q24	*“(P10), great point about forming more of a local COP, basically with the two hospitals linking or even maybe the rehab hospital as well and using those resources. Um, so between those three hospitals.” And in the meantime, um, he also offered the research team can probably be great resources for therapists as well.”—P1 (Res)*
b. National context	Q25	*“So, we do have a, as P1 said, an activity-based therapy community of practice... It’s, it’s ABT, so it’s not just about FES, but of course FES falls under the umbrella of ABT. So that there is a structure, I guess, that already exists for a community of practice that perhaps FES cycling would align with quite well if that was something down the road that you’ve, you felt was appropriate…We do have regular meetings where members attend, can pose questions, and dialogue with one another.” –P8 (Res)*
4. Infrastructure		
a. Training system and role identification	Q26	*“If you don’t have a system for training and how to do that, I think things tend to get left often and die away from well-intentioned people trying to put things into practice.” -P5 (Res)*
	Q27	*“So, there are different roles that can be useful at each stage, and each stage doesn’t require all of the knowledge of the considerations and the parameters.”—P4 (Admin)*
	Q28	*“After our training session that we had in person, that really helped me implement it with my first patient. But even so, I still needed mentorship from someone else that had experience with it. Just using it on my very first patient.”—P7 (OT)*
b. Bridging the gap from acute care to the community	Q29	*“I think that the continued use and follow up is, is really important for the benefits of FES cycling. So at least having something to bridge that gap from acute care to wherever they’re going after that would be great.”—P6 (PT)*
	Q30	*“going back to the earlier point about transition, it’s critical so that the clinicians and patients know that FES cycling can be continued after they leave acute care, so in the rehab or community.”—P1 (Res)*
c. Sustainability	Q31	*“I do like the idea of keeping it not-for-profit and free, especially for patients, but for sustainability, I wonder if there’s something to explore with the [name] company as well, because it’s their bike, and maybe they want to fund some of this too, right?”—P6 (PT)*
	Q32	*“It’s just maybe donation, like you know, it can put like, uh, “Just please consider donation to keep these resources for free.” And maybe you raise a little bit of money in the course of two years for being hosting and a few expenses. And then if, at some point, it needs to be updated and more work needs to be put in it, then pay it for someone, maybe, I know a research project could be funding it or some grants if really somebody wants to look into it.”—P11 (PLEx)*
	Q33	*“For sustainability, I wonder if there’s something to kind of explore with the [vendor] as well, because it’s, it’s their bike, and maybe they wanna fund some of this too, right?”—P7 (OT)*
	Q34	*“So we [are] actually lacking a so-called a real champion, someone that who has real experience. And I totally understand stuff, like, I would love to be that person, but I need to see more patients so that I can support my staff and because (P7) is already quite busy with the caseload on the units. And my only worry is when people say, “Oh, that should be working and it should be started at acute care and we just don’t have staff to do it.” And how can we support that?”—P10 (PT)*

PT, physical therapist; PLEx, person with lived experience; Admin, administrator Re- researcher; OT, occupational therapist.

### 3.1. Health care provider toolkit categories and content

This FES cycling toolkit is designed specifically for HCPs, including allied health, therapy assistants and nursing, who work with hospital patients who have an acute SCI. This category describes the different headings and information provided within the HCP toolkit.

#### 3.1.1. Overall considerations.

Within the HCP toolkit, participants wanted to consider different people involved with FES cycling delivery. These people include clinicians who make decisions about selecting FES cycling, assistants who run the session, and students who complete the hardware setup (Q1). Participants noted that each toolkit should be tailored to one group and contain different categories (Q2).

#### 3.1.2. Introduction.

Focus group members found it useful to have an introduction to provide context at a high level (Q3). They also wanted rationale for staff incorporating FES cycling into acute care (Q4) in addition to clear, standardized wording about evidence-based expectations about SCI recovery related to FES cycling (Q5).

#### 3.1.3. Benefits and risks.

Like the patient toolkit, participants wanted to include benefits and risks within the HCP toolkit (Q6).

#### 3.1.4. Bike login, setup, and modifications.

Participants commented it would be helpful to have information regarding bike setup (e.g., seat height) (Q7). They also wanted to include setup logistics (e.g., bike login) (Q8).

#### 3.1.5. Dosage parameters, muscle selection, and outcome measures.

A cheat sheet for dosage parameters was suggested as part of the toolkit (Q9). Additionally, participants suggested including muscle selection and how to facilitate decisions about parameter selection (Q10). Focus group members also wanted outcome measures to promote standardized data collection and trials (Q11).

#### 3.1.6. Patient screening.

Participants wanted to include precautions, contraindications, and indications (Q12) within the HCP toolkit ensuring medical stability, looking at the physical function and emotional readiness of the whole patient (Q13).

#### 3.1.7. Before, during, and after the FES cycling session.

Focus group members wanted a before and after checklist for HCPs that provides content for the nurses and care team, such as checking vital signs and skin checks (Q14, Q15). The checklist should include what to check for during the session, immediately afterwards, and the following day (Q16).

#### 3.1.8. Glossary.

The glossary for HCPs should include FES-related terminology only, according to the focus group (Q17). HCPs should already understand other medical definitions as part of their training.

### 3.2. Health care provider toolkit end product

This category describes focus group suggestions for the FES cycling toolkit end products for HCPs.

#### 3.2.1. PDF.

Focus group members discussed a toolkit PDF. They suggested that PDFs are easily shared and posted (Q18). A PDF is useful because links to multimodal aspects are embedded, and it is interactive (Q19). For example, the linked PDF can connect to a quiz to test your knowledge or video demonstration.

#### 3.2.2. Website.

Like the patient toolkit, participants noted that a website with multimedia links would provide enough information (Q20). They did not perceive a need for an app, stating that most HCPs do not have access to work phones (Q21). They found it unlikely that most HCPs would download an app on their personal phone. Participants also suggested including visual info, pictures, videos, and case examples (Q22).

### 3.3. Collaboration between groups and institutions

This category describes the potential opportunities for collaboration between groups (e.g., allied health with FES cycling experience) or institutions (e.g., community of practice formed among hospitals). These collaborations spanned from the local context in Edmonton to the national context across Canada.

#### 3.3.1. Local context.

Participants wanted to consider a soft launch like sending the toolkit to local therapists in Alberta with FES cycling experience (Q23). They also suggested forming a local community of practice with the two acute care hospitals and potentially the rehabilitation hospital with the research team acting as a resource (Q24).

#### 3.3.2. National context.

Focus group members suggested expanding the toolkit across Canada in the future. They mentioned using the existing structure already in place for an ABT Community of Practice because FES falls under the umbrella of ABT (Q25). The Community of Practice has regular meetings where members pose questions and dialogue with one another.

### 3.4. Infrastructure

This category describes the components needed to implement the FES cycling toolkit within and beyond the acute care hospital system. Also, the characteristics of the toolkit needed to promote long-term sustainability are discussed as part of the infrastructure.

#### 3.4.1. Training system and role identification.

Participants noted the importance of a training system. Otherwise, they suggested that tasks related to implementation might be neglected despite good intentions (Q26). Different roles could be useful at each implementation stage, including mentorship from an experienced FES cycling user (Q27). Mentorship helped in addition to in-person training sessions (Q28).

#### 3.4.2. Bridging the gap from acute care to the community.

According to focus group members, continued use and follow up (i.e., bridging the gap) is important to maintain the benefits of FES cycling (Q29). Facilitating transitions in care are critical so that clinicians and patients know they can continue FES cycling after acute care (Q30).

#### 3.4.3. Sustainability.

Participants wanted to keep the toolkit not-for-profit and free, especially for patients (Q31). They suggested having users consider a donation (Q32). In addition to research funding or grants, this strategy would support hosting, or updates. For sustainability there may be something to explore with the FES cycle vendors as well (Q33). Focus group members also expressed concerns that they lack a “real champion”, and unit caseloads are already busy (Q34). They wanted to ensure enough staff participated in the implementation efforts to sustain FES cycling in acute care.

## 4. Discussion

According to our inductive content analysis, there were four main categories identified in the focus groups informing the development of an FES cycling toolkit for SCI rehabilitation in acute care. The first two categories were (1) Health care provider toolkit content and categories (Subcategories: Overall considerations, introduction, benefits and risks, bike login, setup and modifications, dosage parameters, muscle selection and outcome measures, patient screening, before, during and after the FES cycling session, and glossary) (2) Health care provider toolkit end product (Subcategories: PDF and website). The other main categories were (3) Collaboration between groups and institutions (Subcategories: National context and local context) and (4) Infrastructure (Subcategories: Training system and role identification, bridging the gap from acute care to the community, and sustainability). Our results culminated in the design of an acute care toolkit for FES cycling for SCI rehabilitation that can be accessed at www.fescyclingtoolkit.com [39].

### 4.1. Toolkit target audiences, content and categories

Previous educational toolkits have been developed as part of SCI practice [40–42]. Many of these toolkits provide information about assessments and outcome measures for clinicians working with people with SCI. In contrast, our toolkit provides information specific to an FES cycling intervention for acute SCI rehabilitation. These SCI-related toolkits have been created by researchers in collaboration with clinicians. When creating an intervention toolkit, it is also important to include end product users as part of the development phase. For example, when co-designing the SCI Get Fit toolkit, Arbour-Nicitopoulos et al. consulted SCI consumers, researchers, HCPs and community service providers [43]. Executives and staff from SCI community-based organizations, people living with SCI, students, and researchers co-constructed a tool to assess outcomes of peer support programs [44]. Similarly, a toolkit to improve primary care for persons with SCI was developed by Milligan et al. using the opinions of family physicians, rehabilitation specialists, and non-physician clinicians [45].

For our project, end users including individuals with lived experience and health care providers, discussed the development of the toolkit through PAR research [30]. While there are parallel websites such as SCIRE Professional [46] and SCIRE Community [41], we have not identified any dual toolkits that target people with lived experience, their caregivers, and HCPs. According to Gustavsson et al., family and caregiver support are essential when trying to include evidence-based practice as part of a therapy program [47]. Importantly, both HCPs’ and patients’ preferences, values, and individual beliefs impact the implementation of evidence-based practice. Vincenten et al. and Leeman et al. note that this underscores the need to tailor resources to the correct audiences is an essential component of implementation [48,49].

The content and categories for the FES cycling toolkit reflect the perspectives of focus group members. Not only did we develop the toolkit using an evidence-based research design with key interest groups, but we also presented research evidence within it. For HCPs, barriers to evidence-based research implementation include a lack of time and/or confidence to summarize and review research [50]. The evidence-based information provided within our toolkit was curated and synthesized. Furthermore, it enables HCPs to scan the literature instead of engaging in time-consuming searches or reading lengthy systematic reviews. For the patient, the toolkit provides accurate information when they may not have the skills to assess online search results [51]. Also, patients may have limited medical vocabulary and literacy affecting their interpretation of terminology [52,53]. This may result in misinterpretation of benefits and risks associated with FES cycling [54,55]. By linking medical concepts to simplified definitions through the hover function, our toolkit addresses a potential barrier to understanding medical terms. Additionally, we provided a glossary with FES cycling-related definitions.

Like our results, Sander et al. reported that clinicians who are new to using FES may have difficulty knowing where to start [50]. Fewer studies document parameters for the search terms *FES cycling + acute SCI* (*n* = 44) compared to *FES cycling + chronic SCI* (*n* = 73) according to a PubMed search on 20 June 2024. In addition to a parameter section, for the HCP toolkit, we included a glossary with FES-specific terminology to support new clinicians, or those who have not recently delivered FES cycling.

### 4.2. Toolkits as end products to facilitate the uptake of rehabilitation research evidence and technology

Our toolkit end product addresses limitations and suggestions for increasing the uptake of evidence-based treatments identified by Sander et al. [50]. Access to scientific resources is often cited as a barrier to evidence-based practice for HCPs and people with lived experience [56]. The FES cycling toolkit addresses this barrier by providing users with Open Access. It is a free resource that provides users with access to a summary of the evidence and full re-use rights [57].

The toolkit is presented through a website that includes a PDF and video according to participants’ preferences. In 2016, Hammar et al. noted that 89% of respondents read a printed patient information leaflet and 41% reacted positively towards an electronic version (i.e., PDF) [55]. At this time, participants preferred the paper version and indicated concerns about older adults’ computer literacy. However, research by Beaudoin et al., published in 2020, shows that an outcomes toolkit website for SCI tracked almost 190,000 users across six years [58]. With the movement away from hard copies towards online storage and increased computer use, accessibility has also improved. For example, with our toolkit, users can enlarge the font for both the website and electronic PDF. They can also use widely available, free software to translate the toolkit to other languages and convert text-to-speech. We provide bulleted lists and printable checklists throughout the website and PDF similar to fact sheets suggested by Sander et al. This also avoids long sentences that may be a barrier to use. Despite the advantages of a website and electronic PDF, it may be useful for HCPs to provide a printed copy for patients to provide options for access and increase dissemination [59].

A video can increase an individual’s understanding of evidence-based information [60]. Chatterjee et al. identified positive results in 48 out of 65 studies from a video intervention used to educate patients [61]. Similarly, a scoping review by Dahodwala et al. demonstrated that video-based education had a significantly positive effect on knowledge, clinical/physical, mental/emotional, and behavioural outcomes [62]. Further, this type of education increased the chance patients would become active participants in decision making [63]. Video-based education also improved short-term outcomes such as understanding medical information, improving patient satisfaction, and reducing anxiety [62]. Our patient toolkit included an educational video to demonstrate what an FES cycling session would entail. We used a “Professionals in Practice” format which shows people engaged in specific practices, such as using FES cycling. Research has shown the impact of different educational video formats is similar across patient outcomes [62].

### 4.3. Collaboration between groups and institutions

An organizational culture with engaged managers, clinicians, and departments helps with evidence-based practice adoption [50]. To develop this toolkit, we had local support from different administration levels at two urban acute care hospitals in Edmonton, Alberta. We included allied health HCPs from these hospitals within the focus groups and also presented the draft toolkit at these sites. A researcher, clinical champion, and an individual with lived experience gave these presentations to staff and HCPs. Allied health HCPs who work in acute stroke rehabilitation attended due to their use of FES cycling with this population as well.

In addition to local support, partnering with professional organizations contributes to moving research to practice [50]. National groups such as the Canadian ABT Community of Practice and Praxis Spinal Cord Institute have hosted presentations about the FES cycling toolkit. Provincially, the Alberta FES Interest Group has also held an invited talk on the same topic. These strategies will increase the profile of this resource for numerous stakeholders both locally and nationally.

### 4.4. Infrastructure

The toolkit is one component of a training system to implement FES cycling within acute care hospitals. Within the system, clinical champions and other HCPs who provide support and mentorship can act as bridges between the technology and its use [50]. We identified and recruited clinical champions early as part of our study. We also educated clinical staff about the toolkit. Other parts of a training system could include initial and ongoing hands-on training that are already available. Like Brockman et al., we used broad, feasible strategies [i.e., conduct educational meetings, prepare consumers for active participation) to begin implementation of the toolkit and FES cycling [64]. Other strategies, spanning the implementation categories mapped by Waltz et al. [e.g., change physical structure and equipment, fund and contract for the clinical innovation) may be more effective, but would require further analysis [65]. Moving forward, individualized implementation strategies should be considered for each FES cycling facility following expert recommendations [66]. A comprehensive implementation plan would facilitate sustainability across sites.

### 4.5. Study limitations and future directions

Future research could include toolkit expansion to chronic SCI or modification for people with other neurological conditions [e.g., stroke, multiple sclerosis) who may use FES cycling for rehabilitation. We could consider other regions across Canada and expand into the United States and other countries. The toolkit should be considered as part of a comprehensive implementation strategy for FES cycling. Next steps should also evaluate the effectiveness of the toolkit in facilitating FES cycling in acute care. A limitation of this study was that we did not include caregivers within our focus groups; however, we could include them as we expand the toolkit further. Furthermore, we have only included academic acute care rehabilitation settings. Non-academic settings face different challenges that could be included in a future toolkit edition as well.

## 5. Conclusions

Our toolkit resource is applicable to individuals living with SCI and HCPs who work in acute care in urban hospitals. However, the information can also support FES cycling for SCI rehabilitation at other acute care hospitals, rehabilitation sites, and community centres in Canada. The content can inform the use of FES cycling for other neurological conditions such as multiple sclerosis and stroke.

## Supporting information

S1 Appendix Interview guide. (DOCX)

S2 Appendix Usability survey questions. (DOCX)
