# Supplementary material for: Development of a functional electrical stimulation cycling toolkit for spinal cord injury rehabilitation in acute care hospitals: A participatory action approach
Source: PLoS One. 2025 Feb 10;20(2):e0316296. doi: 10.1371/journal.pone.0316296 (PMC11809891; doi:10.1371/journal.pone.0316296)
Supplement: S1 Appendix — (DOCX) [file pone.0316296.s001.docx]

# Supporting information

## Appendix S1. Interview guide

Audio recording will begin now.

Before I begin, I would like to reiterate that this one-hour focus group will be recorded. You can turn off your camera at any time. If you use names or any other identifying information, it will be removed from the transcripts. You can refuse to answer any question. Please indicate that you are still willing to consent to the interview by giving your verbal consent now. (Moderator asks each individual for a response)

FIRST MEETING ONLY: I want to start by giving you a little background information about this project. We are conducting group discussions with people who have a spinal cord injury (SCI), their loved ones, rehabilitation therapists, clinicians, and researchers to learn more about functional electrical stimulation (FES) cycling in the acute care hospital setting. We want to get your opinions so that we can develop an information toolkit. We will use this toolkit to support rehabilitation therapists as they apply FES cycling to therapy for people with SCI. This toolkit will also have information for individuals participating in FES cycling in acute care and their loved ones.

I would like to go over some quick ground rules before we start:

1. Only one person should speak at a time. This is important for the transcription of today’s meeting because it is difficult to capture more than one voice at one time. To help with this, I will moderate the discussion, but also you can use the “raise hand” function so that I will ask for your input.
2. This is a confidential discussion. I encourage all of you to openly share your opinions as you are comfortable.
3. Please let me know if you need a break at any time.
4. Are there any questions before we begin?

In this first meeting, we would like to get to know each other and listen to you present your work that applies to FES cycling in acute care. We would also like to discuss your experiences with FES cycling.

Before we begin, please introduce yourself and your role as it relates to FES cycling in acute care. (Moderator indicates each speaker in turn).

Let’s go around again with each person presenting their relevant work and/or experience as it relates to FES cycling. Is there someone who would like to go first? (Moderator indicates each speaker in turn)

1. What information should we include in the toolkit? (Probe…Equipment information, FES parameters, contraindications) [moderator/note taker writes the list of equipment in the chat box]

Prior to the next meeting, please reflect on what information you think should be part of the FES cycling toolkit in acute care. Also, think about the order of importance of each category of information and what format the overall toolkit should take.

SECOND MEETING ONLY: In this meeting, we would like to discuss toolkit categories further and content that we should include. We should also rank the categories in order of importance. For the last item, we will decide what format the toolkit should take.

1. Is there any information that was not mentioned last time that we should include in the toolkit? [moderator/note taker writes the original list of equipment in the chat box and adds new suggestions]
2. Should we have one toolkit that includes all categories, or should we have two toolkits, or more, one targeting “health care providers” and another targeting “patients and social supports”?
3. Are there any categories that should or should not be provided in a “health care providers” toolkit versus a “patients and social supports” toolkit?
4. Let’s prioritize the toolkit information [or Let’s prioritize the toolkit information for the “health care provider” toolkit (followed by) Let’s prioritize the toolkit information for the “patients and social supports” toolkit]. For (category) please indicate a number out of (total categories) in a private chat to me. (Continue in this manner until each category has a number from each focus group member)
5. What format should the toolkit take? (Probe: website, online manual, embedded audio/video in the manual, app)

THIRD MEETING ONLY: In this meeting, we would like to discuss marketing and ways to distribute the toolkit. We would also like to look through the draft toolkit and get any feedback. (Focus group members will be provided a copy of the draft toolkit at least one week prior to this meeting)

1. Let’s go through the toolkit page by page. As we look at each page, please let me know what you think.
2. Now that we have feedback for the draft toolkit, let’s discuss how we can market the toolkit to our target audience(s). (Probe: online platforms, in person, hard copies)

FOURTH MEETING ONLY: I am going to share the website so that I can click through it to the appropriate section as you provide your feedback. You can also pull it up on your screen at [www.fescyclingtoolkit.com](http://www.fescyclingtoolkit.com). (Moderator shares FES cycling toolkit website on screen)

I provided you with the APEASE criteria in the meeting reminder for Focus Group #4. I have also put it in the chatbox. APEASE stands for Acceptability, practicability, effectiveness, affordability, spillover effects and equity of an intervention. In this case, the FES cycling toolkit is the intervention.

**(Moderator places in chat box):**

**Acceptability**: how far the FES cycling toolkit is likely to be liked or engaged with.

**Practicability**: how far the FES cycling toolkit can or is likely to be able to be delivered as planned and at the scale intended. [The intended scale is for Health Care Providers, People with Lived Experience and their supports who have provided or undergone care at the University of Alberta or Royal Alexandra Hospital in Edmonton, Alberta]

**Effectiveness**: how far the FES cycling toolkit achieves or is likely to achieve a desired outcome [improve knowledge about FES cycling] and provides value for money.

**Affordability**: how the FES cycling toolkit can or is likely to be implemented within an available budget.

**Spillover effects**: how far the FES cycling toolkit has or is likely to have unintended positive or negative effects.

**Equity**: how far the FES cycling toolkit affects or is likely to affect inequalities.

1. Something that is also an important consideration for the FES cycling toolkit is the sustainability of the toolkit. Does anyone have any ideas about how to ensure that people continue to be aware of the toolkit and use it as needed? (Probe: What about using it to train health care providers new to FES cycling? Educating patients and their supports?) (Probe: We talked about promotion before, does anyone have ideas about further promotion?)

ALL MEETINGS:

We want to thank you for your time today. Is there anything that you would like to add before we end the discussion? If you would like to read a transcript of the focus group meeting, it can be emailed to you. You can review it and then let us know if you have any requested additions, deletions, or revisions to make. Thank you.
