# Supplementary material for: Development of a functional electrical stimulation cycling toolkit for spinal cord injury rehabilitation in acute care hospitals: A participatory action approach
Source: PLoS One. 2025 Feb 10;20(2):e0316296. doi: 10.1371/journal.pone.0316296 (PMC11809891; doi:10.1371/journal.pone.0316296)
Supplement: S2 Appendix — (DOCX) [file pone.0316296.s002.docx]

# Supporting information

## Appendix S2. Usability survey questions

Thank you for agreeing to complete this survey on the usability of the Functional Electrical Stimulation Cycling for Spinal Cord Injury Rehabilitation Toolkit Website. Your answers will be anonymous, and you can choose to withdraw from the survey at any time without penalty. It should take you between 5 to 15 minutes to complete.

1. Please indicate which group you belong to:

Person with lived experience or caregiver

Health care provider (e.g., physiotherapist, nurse, therapy assistant)

Administrator

Other (Please specify)

Please indicate to what extent you agree with each statement*.

1. I think that I would like to use this system frequently.

1 – Strongly Disagree

2

3

4

5 – Strongly Agree

1. I found the system unnecessarily complex.
2. I thought the system was easy to use.
3. I think that I would need the support of a technical person to be able to use this system.
4. I found the various functions in this system were well integrated.

1. I thought there was too much inconsistency in this system.
2. I would imagine that most people would learn to use this system very quickly.
3. I found the system very cumbersome to use.
4. I felt very confident using the system.
5. I needed to learn a lot of things before I could get going with this system.
6. Do you have any other comments about the website usability that you would like us to take into consideration?

*Participants rated questions 2-11 on a 5-point Likert Scale, with 1 representing “Strongly disagree” and 5 representing “Strongly agree”. The Likert Scale numbers 2-4 did not have any qualifiers attached to them.
